# A Map of the Initiatives That Harmonize Patient Cohorts Across the World

**DOI:** 10.3389/fpubh.2021.666844

**Published:** 2021-07-15

**Authors:** Ángel Rodríguez-Laso, Laura Alejandra Rico-Uribe, Christine Kubiak, Josep Maria Haro, Leocadio Rodríguez-Mañas, José Luis Ayuso

**Affiliations:** ^1^Thematic Area for Frailty and Healthy Ageing of the Network of Biomedical Research Centers (CIBERFES), Instituto de Salud Carlos III, Madrid, Spain; ^2^Centro de Investigación Biomédica en Red de Salud Mental, Instituto de Salud Carlos III, Madrid, Spain; ^3^European Clinical Research Infrastructure Network (ECRIN-ERIC), Paris, France; ^4^Parc Sanitari Sant Joan de Déu, Barcelona, Spain; ^5^Biomedical Research Foundation, Hospital Universitario de Getafe, Madrid, Spain; ^6^Geriatric Department, Hospital Universitario de Getafe, Madrid, Spain; ^7^Department of Psychiatry, Universidad Autónoma de Madrid, Madrid, Spain; ^8^Instituto de Investigación Sanitaria Princesa, Hospital Universitario de La Princesa, Madrid, Spain

**Keywords:** clinical cohort, patient cohort, harmonization, integration, repository

## Introduction

Integration of cohort studies allows taking advantage of already collected information to increase the sample size to study uncommon exposures, rare diseases, less strong associations, or very restricted populations (personalized medicine). It also allows to carry out standardized analyses and avoid publication bias compared to the analysis of published data (1–5). Nevertheless, the growing energy spent in conducting cohort studies across the world in the last decades has not been paralleled by an effort to make them accessible to the scientific community and harmonize their data. This last limitation moved the European Commission to fund the SYNergies for Cohorts in Health: integrating the ROle of all Stakeholders (SYNCHROS) coordination and support action, endowed with almost €2 million^1^ from 2019 to 2021. It aims “to establish a sustainable European strategy for the development of the next generation of integrated cohorts, thereby contributing to an international strategic agenda for enhanced coordination of cohorts globally, in order to address the practical, ethical, legal, and methodological challenge of optimizing the exploitation of current and future cohort data, toward the development of stratified and personalized medicine as well as facilitating health policy.”

In order to achieve its objectives, the first activity proposed in SYNCHROS was to map the population, patient, and clinical trial cohort integration landscape. That would allow the project to have a first look at the challenges and tried solutions adopted by different groups, and, more importantly, it would provide a list of principal investigators of these initiatives who could be contacted for the process of developing the common strategy. This study reports the result of the mapping of the initiatives that integrate patient cohorts. The mapping of population cohorts will be reported elsewhere. The aim of the study was to obtain a non-exhaustive, but representative, list of these initiatives carried out in recent times in the world. To our knowledge, there is no other repository of integration initiatives of patient cohorts. Although excellent single cohort repositories exist, like the Maelstrom catalog, repositories of initiatives that integrate several patient cohorts could not be found.

This mapping will provide researchers with a useful tool to find initiatives on their areas of interest with whom they can share or analyze harmonized data.

## Methods

The initiatives included in the mapping were obtained from three different sources:

1. Systematic searches, carried out in MEDLINE and the Maelstrom catalog.^3^
2. Suggestions of potential initiatives to be included in the mapping provided by partners of the SYNCHROS consortium.
3. References and links provided by the initiatives detected in the two previous sources.

The inclusion criteria were as follows:

a) initiatives that integrated patient, clinical, or disease cohorts;
b) individual patient meta-analysis and mega-analyses; and
c) at least one cohort included in the initiative having information about the sample at two or more points of time (at least two waves).

The exclusion criteria were as follows:

a) initiatives that only integrate population cohorts or clinical trials, without including patient cohorts;
b) initiatives published before the year 2000; and
c) initiatives that did not provide information in English.

### Database Searches

#### MEDLINE Search

The process started with searches restricted to papers published in English from 2000 to 2019 using the terms selected by consensus among the SYNCHROS partners. Those terms which obtained fewer than 500 hits were retained, and the abstracts of the hits were reviewed to find new terms that were used in subsequent searches. In some cases, the term “cohort” was added to these searches to limit the number of hits.

The final search strategy used is given as follows:

(cohort OR “prospective study” OR “longitudinal study” OR “individual meta-analysis”[All Fields] OR “individual participant data meta-analysis”[All Fields] OR “individual patient data meta-analysis”[All Fields] OR “individual meta analysis”[All Fields] OR “individual participant data meta analysis”[All Fields] OR “individual patient data meta analysis”[All Fields] OR “meta analysis using individual”[All Fields] OR “meta-analysis using individual”[All Fields] OR “meta analysis of individual”[All Fields] OR “meta-analysis of individual”[All Fields] OR “mega-analysis”[All Fields] OR “mega analysis”[All Fields])

AND

(“harmonization study” OR “integration study” OR “integration initiative” OR “integrated study” OR “merged cohort” OR “data pooling” OR “pooled sample” OR “combined data” OR “combining data” OR “harmonized data” OR “harmonised data” OR “harmonizing data” OR “data harmonization” OR “data harmonisation” OR “data sharing” OR “common database” OR “multiple cohorts” OR “multiple longitudinal studies” OR “international consortium” OR “collaborative effort”).

AND

(“2000/01/01”[Date - Publication]: “2019/07/31”[Date - Publication])

AND

English[Language]

AND

Humans[MeSH]

#### Maelstrom Catalog

The Maelstrom research catalog, supported by the Research Institute of the McGill University Health Centre, “contains comprehensive information about epidemiological research networks and studies, and the data they have collected. It also provides information about harmonized data generated by these research networks.”

We looked for initiatives included in the “Networks” section of the catalog.

##### Selection of Initiatives

Initiatives that were obtained from the systematic searches and provided by the partners were evaluated against the inclusion and exclusion criteria by two different investigators. In case of a disagreement, a third reviewer was consulted.

##### Extraction of Information

The following information was extracted from each initiative: name of the initiative, principal investigator, partners, name of the institution responsible for the initiative, funding resources, contact person, information source, whether the research team is currently active, main objectives, criteria for the cohorts to be included in the initiative, type of harmonization (prospective/retrospective), number of cohorts included in the initiative (the total number and the number of harmonized cohorts), whether more cohorts are foreseen to be harmonized, number of participants (the total number and the number of participants with harmonized data), age range of the sample, threats to representativeness of the sample, maximum number of variables that have been harmonized, including those where harmonization was not possible for all the cohorts, setting of the harmonized cohorts (local-regional/national/international, including country of origin of the cohorts), and a brief description of the population considered by the initiative.

All this information was retrieved from the webpage and/or the scientific article that presented the initiative. Missing information was requested from the principal investigators of the projects, who were contacted initially by email and, if there was no answer, by phone call or by post.

## Analysis

**Figure 1:**
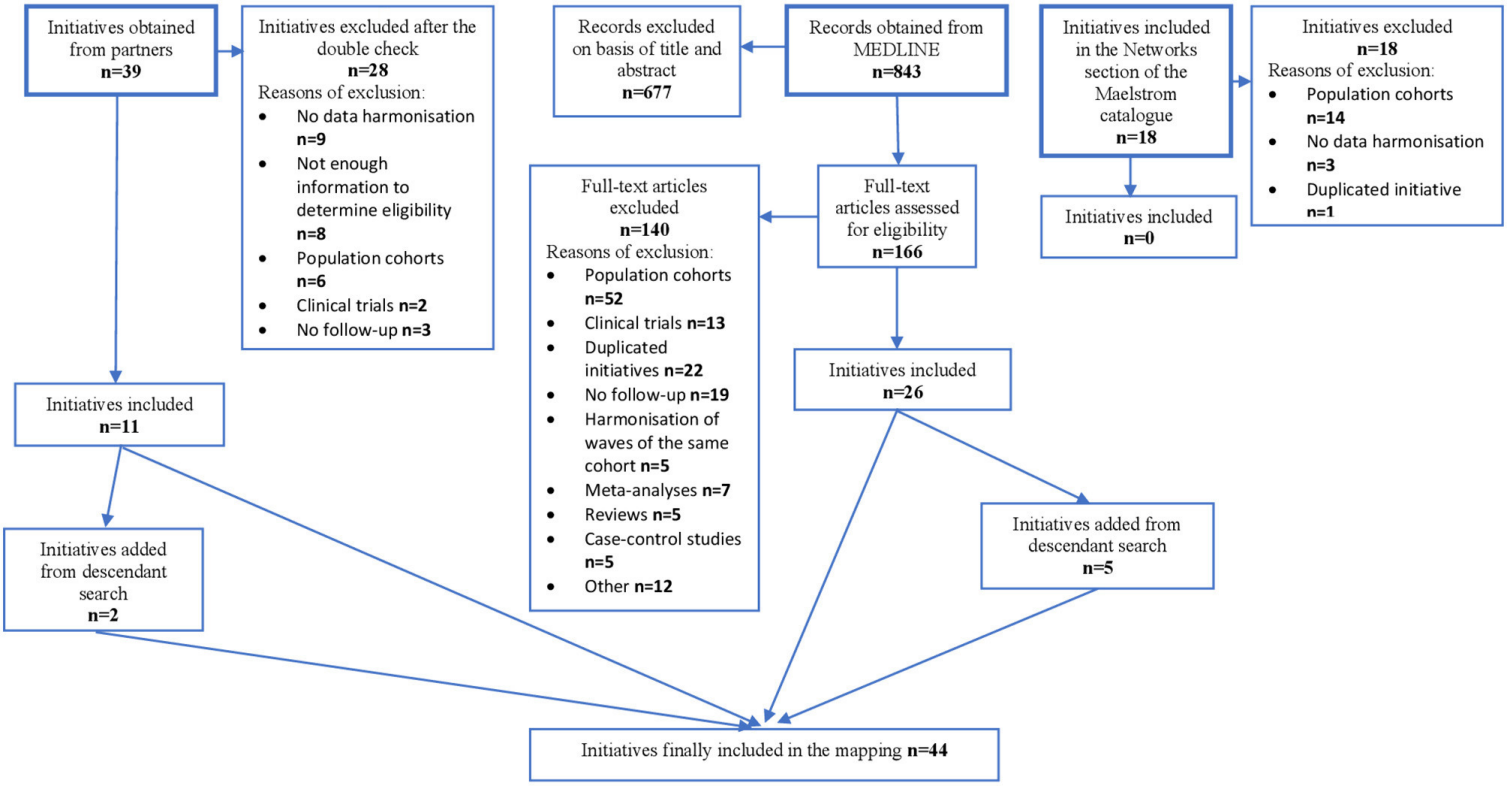
Results of the search for harmonization initiatives of patient cohorts.

Results of the identification process of the initiatives are presented in Figure 1.

Partners of the SYNCHROS project provided 39 initiatives. Of those, 28 were excluded, mainly because there was no data harmonization or because eligibility could not be ascertained due to unresponsiveness from the principal investigators. The remaining 11 initiatives were selected. The descendent search from these initiatives provided two additional ones.

In the MEDLINE search, out of 843 hits obtained, 677 were excluded after reading their title and abstract. Of the remaining articles, 166 were read and, from those, 140 excluded. The main reasons for exclusion were that initiatives dealt only with population cohorts, that they had already been submitted by partners or already presented in another reference, or that the integration was only cross-sectional. In the end, 26 initiatives were selected. The reference list of these initiatives included five additional ones.

The search in the Maelstrom catalog only provided initiatives that harmonized population cohorts.

Overall, 44 initiatives were retrieved. They are presented in Table 1.

**Table 1:** Initiatives that harmonize patient cohorts ordered by different categories of diseases (selected information).

**Initiative**	**Types of cohorts**	**Region, Country were the cohorts were collected**	**Main objective**	**Number of cohorts with harmonized data**	**More cohorts foreseen to be harmonized?**	**Number of participants with harmonized data**	**Age range of the sample**	**No. of harmonized variables (Maximum)**	**Population**
									
CINECA: Common Infrastructure for National Cohorts in Europe, Canada and Africa	Disease cohorts. Population cohorts	Africa, Canada and Europe	To develop a federated cloud enabled infrastructure to make population scale genomic and biomolecular data accessible across international borders, to accelerate research, and improve the health of individuals across continents	In progress	Possibly	In progress	Birth to old age	In progress	The dataset provides a diverse representation of studies in rare disease, common disease and national cohorts over time (longitudinal)
CNODES: the Canadian Network for Observational Drug Effect Studies	Disease cohorts	Canada, US and UK	Use collaborative, population-based approaches to obtain rapid answers to questions about drug safety and effectiveness	Depends on the research question	No	Depends on the research question	All ages	Depends on the research question	Population of Canada, UK and US which is prescribed or dispensed drugs
EHDEN: European Health Data and Evidence Network	Disease cohorts	All Horizon 2020 member states and associated countries	Harmonize in excess of 100 million anonymized health records to the OMOP common data model, supported by an ecosystem of certified SMEs, and technical architecture for a federated network		In progress		18	Considerable	European patients aged 18+
MIRACUM: Medical Informatics in Research and Care in University Medicine	Disease cohorts	Seven states of Germany	The spotlight is here on the data integration centers that will be embedded in the hospital IT-infrastructure and will facilitate the collection and exchange of data within the consortia university hospitals. Furthermore, we will elaborate a programme for strengthening medical informatics by extending the academic offer, including new professorships in the field of medical informatics, a novel, innovative master programme and personnel training. The MIRACUM partners have agreed to share data, based on interoperable data integration centers, develop common and interoperable tools and services, realize the power of such data and tools in innovative IT solutions, which shall enhance patient-centered collaborative research as well as clinical care processes, and finally to strengthen biomedical informatics in research, teaching and continued education	11	No information obtained	No information obtained	0 to the highest age of patients	No information obtained	Patients attended in hospitals of seven German states
Sentinel initiative	Disease cohorts	US	Serve as a system to analyze and assess safety risks in FDA-approved drugs and medical products using electronic health data	17	No information obtained	310 million	All ages	No information obtained	Population of US which is prescribed or dispensed drugs
BiomarCaRE: Biomarker for Cardiovascular Risk Assessment across Europe	Disease cohorts. Population cohorts. Clinical trials	Australia, Europe, Israel, Latin America, New Zealand, South Africa, United States	Assess the value of established and emerging biomarkers for cardiovascular risk prediction	4	No information obtained	8.746	No information obtained	No information obtained	Patients with coronary heart disease or at risk of developing it
CADISP: Cervical Artery Dissection and Ischemic Patients	Disease cohorts	Western Europe and Turkey	International Consortium performing research on ischemic stroke in young and middle-aged adults and in particular on cervical artery dissection	No information obtained	No information obtained	No information obtained	No information obtained	No information obtained	Cervical artery dissection and ischemic stroke patients from some Western European countries and Turkey
Development and validation of the AMPREDICT model	Disease cohorts	US	The objective of this study was the development of AMPREDICT-Mobility, a tool to predict the probability of independence in either basic or advanced mobility 1 year after dysvascular major lower extremity amputation	2	No information obtained	200	No information obtained	38	Individuals undergoing their first major lower extremity amputation because of complications of peripheral artery disease or diabetes
ESCAPE-NET: European Sudden Cardiac Arrest network: toward Prevention, Education and NEw Treatment	Disease cohorts. Population cohorts	Czech Republic, Denmark, France, Italy, Sweden, The Netherlands	Aims to study: (1) risk factors and mechanisms for the occurrence of sudden cardiac arrest (SCA) in the population, and (2) risk factors and treatment strategies for survival after SCA on a European scale	No information obtained	Yes	No information obtained	No information obtained	No information obtained	Patients with sudden cardiac arrest
After Breast Cancer Pooling Project	Disease cohorts (one is based on the follow-up of a randomized clinical controlled trial)	China (Shanghai), US	Examine the role of physical activity, adiposity, dietary factors, supplement use, and quality of life in breast cancer prognosis	4	Yes	18.314	20–83	No information obtained	Breast cancer survivors (women). Cancers were diagnosed between 1976 and 2006
B-CAST: Breast CAncer STratification	Disease cohorts	No information obtained	In B-CAST tools will be developed to allow precise identification of the individual risk of breast cancer, the subtype of cancer that is most likely to develop and the prognosis of that particular subtype	No information obtained	No information obtained	No information obtained	No information obtained	No information obtained	Patients with breast cancer
Collaborative Group on Epidemiological Studies of Ovarian Cancer	Disease cohorts	Worldwide	Study risk factors of oavarian cancer	58	No information obtained	31,000	No information obtained	No information obtained	Women with ovarian cancer
GENIE: Genomics Evidence Neoplasia Information Exchange	Disease cohorts	Canada, France, Netherlands, Spain, UK, USA	It is a multi-phase, multi-year, international data-sharing project that aims to catalyze precision cancer medicine	19	Yes	70,000	All ages	No information obtained	Cancer patients treated at multiple international institutions
HARMONY: European Public-Private Partnership for Big Data in Hematology	Disease cohorts. Clinical trials	All Europe	The HARMONY Alliance uses big data technologies to improve the treatment of seven hematologic malignancies	Acute Myeloid Leukemia: 5 patient cohorts. Multiple myeloma: 15 patient cohorts	In progress	11,664 (aims to harmonize between 75,000 and 100,000 anonymized hematologic patients by the end of the funding period)	All ages are considered	It depends on the specific research question	Patients with blood malignancies
International Collaboration of Epidemiological Studies of Cervical Cancer	Disease cohorts	Costa Rica, Denmark, Norway, Sweden, UK, US	Study the effects of hormonal contraceptive use and other factors on the risk of cervical cancer	9	No information obtained	2,109	No information obtained	No information obtained	Women with cervical cancer
MaGIC: Malignant Germ Cell International Consortium	Disease cohorts	No information obtained	Developing more effective treatments for germ cell tumors (GCT) through scientific inquiry	No information obtained	No information obtained	No information obtained	No information obtained	No information obtained	GCT patients all over the world
NCI: National Cancer Institute Cohort Consortium	Disease cohorts. Population cohorts. Clinical trials	Australia, Canada, New Zealand, USA	Foster communication among investigators leading cohort studies of cancer, promote collaborative research projects for topics not easily addressed in a single study and identify common challenges in cohort research and search for solutions	No information obtained	Yes	No information obtained	18+	No information obtained	Breast and colon family cancer patients and their families
Second primary malignancies in thyroid cancer patients	Disease cohorts	France, Italy, Sweden	Evaluate the risk of second cancer and leukemia in patients with papillary or follicular thyroid cancer treated with radioiodine or external beam radiation therapy	3	No	6,841	7–80 (at time of diagnosis of thyroid cancer)	Around 10	Patients with papillary or follicular thyroid cancer
The International CLL-IPI working group	Disease cohorts. Clinical trials	France, Germany, Poland, UK, US	We established an international consortium with the aim to create an international prognostic index for chronic lymphocytic leukemia (CLL-IPI) that integrates the major prognostic parameters	2		1,254	No information obtained	18	Chronic lymphocytic leukemia patients
COASt: Clinical Outcomes in Arthroplasty Study	Disease cohorts	Europe	Describe whether body mass index is a clinically meaningful predictor of patient reported outcomes following primary total hip replacement (THR) surgery	4	No information obtained	4,413	No information obtained	24	Patients receiving primary THR for osteoarthritis
MARC-35: 35th Multicenter Airway Research Collaboration	Disease cohorts	US	Examine the association between the infectious etiology of a child's severe bronchiolitis and the level of serum 25-hydroxyvitamin D (25[OH]D) during severe bronchiolitis, with the severity of this illness, and the subsequent development of recurrent wheezing by age 3 years and combine these clinical and laboratory data to derive the wheezing index that will identify children at higher risk of developing recurrent wheezing by age 3 years	17	No	920	0–1	Thousands	Children age <1 year hospitalized with severe bronchiolitis
COSMIC: Cohort Studies of Memory in an International Consortium	Disease cohorts	The world	Harmonizing shared, non-identifiable data from cohort studies that longitudinally examine change in cognitive function and the development of dementia in older individuals (60+ years).	Data are harmonized on a project-by-project basis, and only subgroups of the member studies contribute to particular projects	Yes	Data are harmonized on a project-by-project basis, and only subgroups of the member studies contribute to particular projects	40–105	Harmonization is done on a project-by-project basis and the number of studies per project varies. For the largest project with 20 studies there are 16 harmonized variables	60+ years old individuals from 29 countries all over the world
Lifebrain: Healthy minds 0–100 years: Optimizing the use of European brain imaging cohorts	Disease cohorts. Population cohorts	Western Europe	Maximize the exploitation of brain imaging cohorts by bringing together studies on how differences and changes in brain age relate to cognitive function and mental health	1 of anxiety and depression patients	No information obtained	2,981	No information obtained	No information obtained	Patients with anxiety or depression
Seasonal plasticity of cognition	Disease cohorts. Population cohorts	Canada, France, US	Test the hypotheses that season has a significant association with cognition, the odds of being diagnosed with mild cognitive impairment or dementia, cerebrospinal fluid Alzheimer disease biomarkers, and the expression of cognition-associated modules of coexpressed genes in the human brain	2	No information obtained	592	No information obtained	No information obtained	Alzheimer disease patients or patients with cognitive disorders visited in tertiary care clinics
HarmonicSS: HARMONIzation and integrative analysis of regional, national and international Cohorts on primary Sjögren's Syndrome (pSS) toward improved stratification, treatment and health policy making disease	Disease cohorts. Clinical trials	Europe, US	To bring together the largest well-characterized regional, national and international longitudinal cohorts of patients with Primary Sjögren's Syndrome (pSS) including those participating in clinical trials, and by taking into consideration the ethical, legal, privacy and intelectual propiety rights issues for sharing data from different countries, to semantically interlink and harmonize them into an integrative pSS cohort structure on the cloud	No information obtained	No information obtained	No information obtained	No information obtained	No information obtained	Cohorts and clinical trials of patients with Primary Sjögren's Syndrome
SABER: SAfety Assessment of Biologic ThERapy	Disease cohorts	US	Understanding the absolute and comparative risks of adverse events of biologic treatments for patients with autoimmune diseases	4	No information obtained	239,806	All ages	No information obtained	Patients with autoimmune diseases who had at least one dispensing of a biologic agent or comparison non-biologic regimen relevant to their autoimmune disease
Thousand Faces of Lupus	Disease cohorts	Canada	Evaluate factors affecting therapeutic approaches used in clinical practice for the management of systemic lupus erythematosus (SLE), in a multicenter cohort	10	No information obtained	1,497	No information obtained	No information obtained	Patients who meet American College of Rheumatology (ACR) criteria for Systemic Lupus Erythematosus
Tumor Necrosis Factor alpha antagonist use and cancer in patients with rheumatoid arthritis	Disease cohorts	Canada and US	Estimate the association between treatment with biologic disease-modifying antirheumatic drugs (DMARDs) and development of cancer in patients with rheumatoid arthritis	3	No information obtained	8,458	65+	No information obtained	Rheumathoid arthritis patients who had been prescribed DMARDs or methotrexate
GEMRIC: Global ECT-MRI Research Collaboration	Disease cohorts	Japan, Western Europe and US. Currently approaching China	Creating a large database of multi-site imaging data and clinical/behavioral/physiological and metadata for analysis of the neural mechanisms and predictors of electroconvulsive therapy-related clinical response	15	Yes	345	19–86	More than a thousand because the initiative includes diagnostic imaging variables	Patients receiving electroconvulsive therapy
Predictors and moderators of cognitive and behavioral therapy outcomes for obsessive-compulsive dissorder	Disease cohorts. Clinical trials	Australia, Canada, Europe, and US	Identify potential factors that affect the outcome of cognitive and behavioral treatments of obsessive-compulsive disorders	8	No	359	18+ (very few over 65)	Around 20	Patients with obsessive-compulsive disorders
Antibiotic treatment and survival of nursing home patients with lower respiratory tract infection	Disease cohorts	The Netherlands and US	Assess the effects of different antibiotic treatment strategies on survival of elderly nursing home residents with lower respiratory tract infections in the United States and the Netherlands, where treatment approaches are quite different	2	No	1,221	70+	Around 40	Elderly nursing home residents with lower respiratory tract infections
ART-CC: Antiretroviral Therapy Cohort Collaboration	Disease cohorts	Western Europe and North America	Estimate prognosis of HIV-1 positive, treatment naïve patients initiating highly active antiretroviral therapy (ART)	No information obtained	No information obtained	No information obtained	No information obtained	No information obtained	HIV-1 positive, treatment naïve patients cohorts from Europe and North America
COHERE: Collaboration of Observational HIV Epidemiological Research Europe	Disease cohorts	Western Europe and North America	Pool and harmonize existing longitudinal data on HIV-positive persons collected across Europe to answer key research questions that, in the era of potent combination antiretroviral therapy (cART), could not be addressed adequately by individual cohorts	No information obtained	No information obtained	No information obtained	No information obtained	Not reported	HIV-infected people residing in Europe
Early Antibiotic Treatment for Pediatric Febrile Urinary Tract Infection and Renal Scarring	Disease cohorts	US	Determine, in a well-characterized sample of children with febrile urinary track infections, whether delay in the initiation of antimicrobial therapy was associated with the occurrence and severity of renal scarring and to determine whether these associations persisted after adjusting for potential confounding factors	2	No	802	2–72 months	No information obtained	Children aged 2–72 months with a urinary tract infection producing fever
HAART and early mortality	Disease cohorts	Brazil and US	Compare the early mortality pattern and the causes of death among patients starting HAART in Brazil and the United States	2	No information obtained	1,774	No information obtained	10	HIV-infected patients
IeDEA: International epidemiology Databases to Evaluate AIDS	Disease cohorts	Africa, Asia-Pacific region, the Central/South America/Caribbean region, and North America	Collect and define key variables, harmonize data, and implement methodology to effectively pool data as a cost-effective means of generating large data sets to address the high priority research questions and streamline HIV/AIDS research	No information obtained	No information obtained	No information obtained	No information obtained	No information obtained	HIV/AIDS patients from Africa, the Asia-Pacific region, the Central/South America/Caribbean region, and North America
ReCoDID: Reconciliation of Cohort data in Infectious Diseases	Disease cohorts	No information obtained	Develop an equitable, accessible, and sustainable model for the storage, curation, and analyses of clinical-epidemiological and high-dimensional sample data collected by infectious disease cohorts in low-and-midle-income countries	No information obtained	In progress	No information obtained	No information obtained	No information obtained	Patients with infectious diseases
RESPOND: International Cohort Consortium of Infectious Disease	Disease cohorts	Australia, Georgia and Western Europe	Build an innovative, flexible and dynamic cohort consortium for the study of infectious diseases, including HIV and people at risk for HIV, as a generic structure for facilitating multi stakeholder involvement	No information obtained	No information obtained	No information obtained	No information obtained	No information obtained	People 18+ at high risk of acquiring HIV and people living with HIV and/or with other infectious diseases or across Europe, South America and Australia
Adults Born Preterm International Collaboration	Disease cohorts	Australia, Canada, Finland, Netherlands, Northern Ireland, Norway, US	Our main aim was to identify factors that either increase or decrease risk of high blood pressure among adults born with very low birth weight	9	No information obtained	1,571 patients and 777 controls	No information obtained	No information obtained	Very low birth weight and very preterm babies who reach adulthood
Necrotizing enterocolitis (NEC) study	Disease cohorts	Austria and The Netherlands	The first aim of the study was to correlate the occurrence of a blood stream infection (BSI) during the early phase of necrotizing enterocolitis (NEC) with intestinal fatty acid-binding protein (I-FABP) levels, as a marker for loss of gut wall integrity owing to mucosal damage, and Interleukin (IL)-8 levels, as a biomarker for the pro-inflammatory cascade in NEC. The second aim of the study was to investigate the relation between the occurrence of a BSI and disease outcome	2	No information obtained	57	24–40 weeks	13	Patients with necrotizing enterocolitis
Recurrent leg venous ulcers study	Disease cohorts	Eastern Australia	Identify risk and protective factors for recurrence of venous leg ulcers	3	Yes	250	26–96	24	Patients with a healed leg ulcer of primarily venous etiology
MARS: Multicenter AVM Research Study	Disease cohorts	Scotland and US	Identify risk factors for intracranial hemorrhage in the natural history course of brain arteriovenous malformations	4	Yes	2,525	No information obtained	13	Patients with arteriovenous malformations
Pulmonary embolism presentation	Disease cohorts (one clinical trial)	Belgium, France and Switzerland	Compare clinical characteristics between women and men with suspected and confirmed pulmonary embolism (PE) and their impact on clinical probability prediction scores and on diagnostic work-up of PE, and to assess whether differences at presentation could account for the increased recurrence rate in men	3	No	3,414	18–98	Around 30	Patients with a clinical suspicion of pulmonary embolism
BIOMAP: Biomarkers in Atopic Dermatitis and Psoriasis	Disease cohorts. Clinical trials	No information obtained	Examine the causes and mechanisms of atopic dermatitis and psoriasis to enable optimal treatments and an individualized therapy scheme for each patient	No information obtained	In progress	No information obtained	No information obtained	No information obtained	Patients with atopic dermatitis and psoriasis

Table 1 shows a selection of the most relevant information obtained from each of the initiatives. Complete information can be found in the repository of the SYNCHROS project.^3^ They are ordered by types of diseases covered (starting with those which consider several diseases) and by alphabetical order. Of the 44 initiatives found, no further information could be obtained from principal investigators in almost half (20) of them.

Eight initiatives (BIOMAP, CINECA, EHDEN, ESCAP-NET, HarmonicSS, HARMONY, Lifebrain, and ReCoDID) have recently started adding cohorts; 21 are led by active research teams; and 12 are adding, or considering adding, cohorts now. Nevertheless, there is plenty of missing information on the activity status of the initiatives.

In the selected initiatives, the most represented group of diseases is cancer (10 initiatives), followed by infectious diseases (8 initiatives, of which 5 focus on HIV) and cardiovascular disease (4 initiatives). There are five initiatives that have harmonized data from more than one type of disease. Other diseases and conditions producing a high burden in the high-income countries (6) are represented (dementia, osteoarthritis), but others included in this list (unipolar depressive disorders, alcohol use disorders, hearing loss, chronic obstructive pulmonary disease, diabetes mellitus, road traffic accidents) or poor-defined conditions with a well-defined impact on life-expectancy and quality of life (like back pain or functional deterioration) are missing. There is one initiative about a specific rare disease (Sjögren syndrome).

There is a sizable number of initiatives that have harmonized other types of cohorts in addition to patient cohorts. After Breast Cancer Pooling Project, BIOMAP, CLL-IPI, HARMONY, and the initiatives on obsessive-compulsive disorder and pulmonary embolism have harmonized at least one clinical trial cohort. CINECA, ESCAPE-NET, Lifebrain, and the project “Seasonal plasticity of cognition” have also harmonized population cohorts. BiomarCaRE and the National Cancer Institute Cohort Consortium have harmonized the three types of cohorts: patient, population and clinical trials cohorts.

Most of them (33) have an international scope, compared to seven national initiatives and one regional/local initiative. Two initiatives report that they include cohorts from across the world and eight initiatives incorporate cohorts from high- and low- and middle-income countries (LMIC); 30 (75%) initiatives only include cohorts from high-income countries, and none harmonize data from LMIC countries alone.

Most initiatives are partnered with universities, hospitals, and research institutes. Governmental institutions take part in a few of them (9). The presence of patient associations and pharmaceutical companies as partners is anecdotal. The number of partners ranges between 2 and more than 100, with a median of 12. Three quarters comprise 20 partners or fewer.

Most initiatives have been or are funded by American (12) or European (10) institutions. Canadian funding comes third (4). The vast majority have received public funding alone (22). Five have received combined funding from public institutions and non-profit organizations. Private funding was provided in isolation to one initiative (RESPOND), combined with public funding to another one (EHDEN), and combined with non-profit funding to a third one (Tumor necrosis factor α antagonist use).

Their objectives may be classified into four general categories (some initiatives share more than one): determining the prognosis of subgroups of patients (14), providing a repository of patients (11), establishing the efficacy (6) or safety (4) of treatments, and exploring risk factors and biomarkers of diseases (10).

The median number of cohorts included in each initiative is 5, ranging from 1 (which also harmonizes population cohorts) to 58; three quarters include 17 cohorts or fewer. The number of individuals included varies wildly, from 57 to 310 million (Sentinel initiative). The median is 6,841. Eight out of 37 (21.6%) initiatives have harmonized fewer than 1,000 patients and the same proportion have harmonized 100,000 patients or more. Twenty-six have harmonized all or almost all the cohorts incorporated to the initiative, two (EHDEN and CINECA) are still in the process of harmonizing their cohorts and another two (CNODES and COSMIC) harmonize data on a project-by-project basis.

Eight initiatives included patients from all ages, eight included only adult patients, three included only children, and two included exclusively older people.

Of those which have declared the number of variables in their harmonized database, there are between 10 and more than 1,000 (median 24), with two out of 15 (13.3%) including more than 1,000 variables.

Four initiatives harmonized administrative databases. Thirty-three were retrospective vs. four prospective. The great majority do not report major threats to the representativity of their samples.

## Data Availability Statement

The dataset generated by this study can be found in the webpage of the SYNCHROS project https://www.synchros.es.

## Author Contributions

ÁR-L performed the database searches, extracted the information from the initiatives, and drafted the article. ÁR-L, LR-U, and CK evaluated the initiatives against inclusion and exclusion criteria. All authors made substantial contributions to the conception and design of the paper, analysis and interpretation of data, reviewed the manuscript, and read and approved its final version.

## Conflict of Interest

The authors declare that the research was conducted in the absence of any commercial or financial relationships that could be construed as a potential conflict of interest.

## Footnotes

^1^https://cordis.europa.eu/project/id/825884/es.

^2^https://www.maelstrom-research.org/maelstrom-catalogue.

^3^https://www.synchros.es.
